# Cholesterol Interaction Directly Enhances Intrinsic Activity of the Cystic Fibrosis Transmembrane Conductance Regulator (CFTR)

**DOI:** 10.3390/cells8080804

**Published:** 2019-07-31

**Authors:** Stephanie Chin, Mohabir Ramjeesingh, Maurita Hung, June Ereño-Oreba, Hong Cui, Onofrio Laselva, Jean-Philippe Julien, Christine E. Bear

**Affiliations:** 1 Programme in Molecular Medicine, Research Institute, Hospital for Sick Children, Toronto, ON M5G 0A4, Canada; 2 Department of Biochemistry, University of Toronto, Toronto, ON M5G 0A4, Canada; 3 Department of Physiology, University of Toronto, Toronto, ON M5G 0A4, Canada; 4 Department of Immunology, University of Toronto, Toronto, ON M5G 0A4, Canada

**Keywords:** membrane protein purification, amphipol:A8-35, intrinsic anion channel activity, catalytic activity, proteoliposomal flux, functional reconstitution

## Abstract

The recent cryo-electron microscopy structures of zebrafish and the human cystic fibrosis transmembrane conductance regulator (CFTR) provided unprecedented insights into putative mechanisms underlying gating of its anion channel activity. Interestingly, despite predictions based on channel activity measurements in biological membranes, the structure of the detergent purified, phosphorylated, and ATP-bound human CFTR protein did not reveal a stably open conduction pathway. This study tested the hypothesis that the functional properties of the detergent solubilized CFTR protein used for structural determinations are different from those exhibited by CFTR purified under conditions that retain associated lipids native to the membrane. It was found that CFTR purified together with phospholipids and cholesterol using amphipol: A8-35, exhibited higher rates of catalytic activity, phosphorylation dependent channel activation and potentiation by the therapeutic compound, ivacaftor, than did CFTR purified in detergent. The catalytic activity of phosphorylated CFTR detergent micelles was rescued by the addition of phospholipids plus cholesterol, but not by phospholipids alone, arguing for a specific role for cholesterol in modulating this function. In summary, these studies highlight the importance of lipid interactions in the intrinsic activities and pharmacological potentiation of CFTR.

## 1. Introduction

Cystic fibrosis is caused by mutations of the cystic fibrosis transmembrane conductance regulator (*CFTR*) gene that codes for the ATP-binding cassette (ABC) anion channel, CFTR [1,2,3]. The CFTR channel activity is regulated by protein kinase A (PKA) phosphorylation of its regulatory (R) domain as well as ATP binding and hydrolysis by its nucleotide binding domains (NBDs) [4,5,6,7,8,9]. Biochemical and electrophysiological studies support a model where phosphorylation promotes dissociation of the R domain from inhibitory locations at the interface between the NBDs to facilitate their dimerization and ATPase activity [7,8,10,11,12,13,14,15,16,17,18]. This model predicts that the NBDs of the unphosphorylated and nonconductive form of CFTR may be dissociated, whereas the NBDs may be dimerized in the phosphorylated and conductive form of the protein.

Recently, six structures of CFTR have been determined using cryo-electron microscopy (EM) at resolutions of 3.2–3.9 Å [19,20,21,22,23]. These structures include the unphosphorylated, ATP-free and phosphorylated, ATP-bound forms of the zCFTR and hCFTR proteins [19,20,21,22,23]. A comparison of the structures shows major conformational changes upon PKA phosphorylation and ATP binding that are consistent with the molecular mechanism proposed for opening of the conduction pore through CFTR. These changes included the loss of stable R domain interactions at the cleft between the two transmembrane domain (TMD)-NBD halves of the CFTR molecule and tighter association of the NBDs [20,21,22]. There were also subtle conformational changes at the transmembrane segments (TMs), notably at TM8 and TM12, upon PKA phosphorylation and ATP binding [20,22]. These structures suggest that both TM8 and TM12 helices rotate to participate in novel interactions upon CFTR phosphorylation [20,22]. Surprisingly, the structure of phosphorylated, ATP-bound CFTR proteins did not reveal a continuous pathway for anion conduction [20,22]. Hence, factors required to stabilize the open conduction pathway may be lacking in the CFTR proteins studied by cryo-EM.

Recent studies have shown that ABC transporters specifically bind to lipids and these protein: lipid interactions, are structurally important [24,25,26]. Phospholipids and cholesterol are bound to ABCG2 in a recent cryo-EM structure [24,25] and conformers of MsbA solved in lipid containing nanodiscs differ from those determined in detergent micelles [26]. Furthermore, lipid interactions are known to modify the activity of several ABC transporters including: P-glycoprotein [27,28], ABCG1 [29], LmrA [30] and MsbA [31,32]. In fact, the addition of lipids was found to stabilize CFTR after its purification in detergent and modulate its ATPase activity [33,34]. Hence, this study developed a novel method to purify CFTR in a detergent-free environment in order to test the modulatory effect of lipids. This study confirmed that, relative to detergent purified protein, CFTR extracted from mammalian membranes using amphipol A8-35 retains associated lipids and these lipid interactions were important for modulating the intrinsic functional properties of CFTR, including ATPase activity, channel function and the response to the potentiator called ivacaftor.

## 2. Results

### 2.1. CFTR: Lipid Complexes Are Purified Using Amphipol A8-35

**Figure 1:**
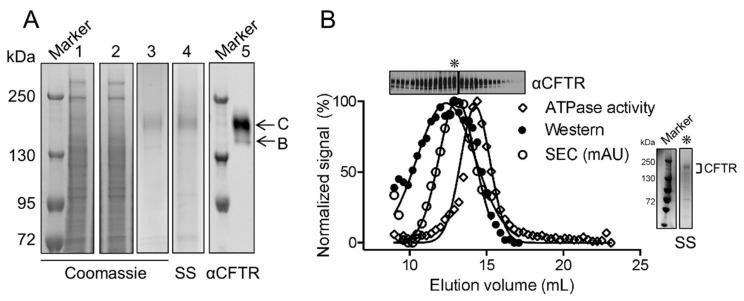
Functional cystic fibrosis transmembrane conductance regulator (CFTR) can be purified using amphipol. (**A**) Protein gels stained by Coomassie blue of lysate solubilized by amphipol (1), unbound fraction (2) and eluate from anti-FLAG^®^ M2 magnetic beads (3). The eluate from anti-FLAG^®^ M2 magnetic beads was also stained by silver stain (SS) (4) and immunoblotted (5) showing that CFTR was effectively purified as a relatively pure population. CFTR appeared as two bands that include a band at approximately 170–180 kDa that represents the mature, complex glycosylated form of the protein (band C) and a band at approximately 140–150 kDa that represents the immature, core glycosylated form of the protein (band B). (**B**) The overlay of normalized signal of CFTR ATPase activity (open diamonds) and CFTR protein detected by immunoblot (closed circles) to the size exclusion chromatography (SEC) trace on a Superose 6 10/300 GL (GE Healthcare) (open circles) showing that the SEC peak (asterisk) corresponds to CFTR protein separation and the shoulder of that peak corresponds to functional CFTR. SS of the SEC peak (asterisk) shows the purified CFTR protein is relatively pure.

The full-length human Wt-CFTR, bearing its native sequence except for two affinity tags, a FLAG tag (on the amino terminus) and a 10× histidine-tag (on the carboxy terminus) was expressed in HEK293F cells. Amphipol A8-35 at 30 mg was effective in solubilizing CFTR from 1 mL of 2 mg/mL final concentration of re-suspended membranes, which corresponds to an amphipol concentration of 3% (*w/v*) (Figure 1A, lane 1). Finally, CFTR was purified to near homogeneity in a single step by affinity to anti-FLAG^®^ magnetic beads (Figure 1A, lane 3 and 4). In SDS-PAGE, purified CFTR migrated as expected for the mature, complex glycosylated band C of the protein (170–180 kDa, Figure 1A, lane 5). The immature, core glycosylated band B of CFTR was also present in the eluted sample as a band at approximately 140–150 kDa (Figure 1A, lane 5).

Size exclusion chromatography (SEC) was used to characterize the purified CFTR protein on the basis of differences in size using 280 nm to monitor the elution of CFTR (Figure 1B). The fractions eluting between the elution volume of 9–17 mL containing both core and complex glycosylated CFTR, as confirmed by silver staining and immunoblotting, co-eluted with ATPase activity (Figure 1B). Interestingly, the peak ATPase activity at elution volume of 14.5 mL was slightly displaced from the peak CFTR protein abundance at the elution volume of 13 mL, suggesting that the population of purified CFTR molecules is heterogeneous with respect to specific enzyme activity.

**Figure 2:**
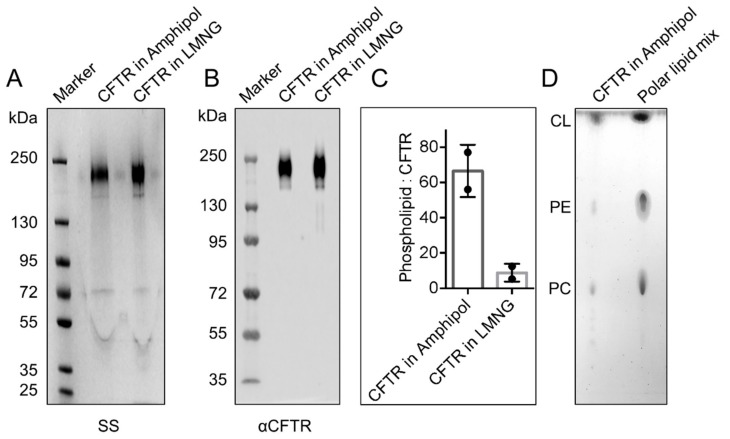
The yield of CFTR is similar for amphipol and Lauryl Maltose Neopentyl Glycol (LMNG) purifications and amphipol purified CFTR retains an annulus of phospholipids and cholesterol. (**A**) SS and (**B**) immunoblot of purified CFTR from amphipol and LMNG purifications showing that both purifications yielded high purity of the CFTR protein at similar yields. (**C**) The phospholipid analysis showing that purified CFTR solubilized in amphipol contained more phospholipids (56 to 76 phospholipids per CFTR molecule) than CFTR solubilized in LMNG (5 to 12 phospholipids per CFTR molecule) after normalizing to protein amounts. The data is presented as a range of *n* = 2 technical replicates. (**D**) The lipid thin-layer chromatography (TLC) showing that extracted lipids from purified CFTR in amphipol include cholesterol, phosphatidylethanolamine (PE) and phosphatidylcholine (PC) as confirmed by similar migration of known lipids from polar lipid mix standard. These lipids were not present in amphipol by itself.

Previous studies found that amphipols do not compete with lipids, which remain associated to the membrane protein: amphipol complexes [35,36,37]. Thus, this study was interested in determining whether CFTR purified using amphipol also retained lipids. The amphipol-based purification to that employed by the Chen group in their cryo-EM studies was also compared [19,20,21,22,23]. First, the authors confirmed that CFTR solubilized in Lauryl Maltose Neopentyl Glycol (LMNG) detergent micelles [19,20,21,22,23] could be purified using the same protocol that the authors developed for the amphipol preparation (Figure 2A,B). Then, the relative degree of phospholipid association was compared in the amphipol- and detergent-based purifications. Choline-containing lipids (i.e., phosphatidylcholine or PC, lecithin, lysolecithin, and sphingomyelin) were quantified using a fluorimetric assay after normalization for CFTR protein abundance (Refer to the CFTR quantification section in Materials and Methods for more details on the method). A higher number of approximately 56 to 76 choline-containing phospholipids associated per CFTR molecule in amphipol were detected compared to approximately 5 to 12 phospholipids associated per CFTR in LMNG detergent micelles (Figure 2C). Notably, the lipids that associated with CFTR in the amphipol purification include cholesterol, PC and phosphatidylethanolamine (PE) as studied by lipid thin-layer chromatography (TLC) (Figure 2D). This study cannot exclude the presence of additional lipids, given the resolution of the solvent system employed for TLC. In fact, it is expected that phosphatidylserine (PS) is also associated, given its dominant presence in the inner leaflet of biological membranes.

### 2.2. CFTR: Lipid: Amphipol Complexes Exhibit Higher Specific ATPase Activity and Higher Functional Reconstitution as Regulated Anion Channels than CFTR: Detergent Complexes

**Figure 3:**
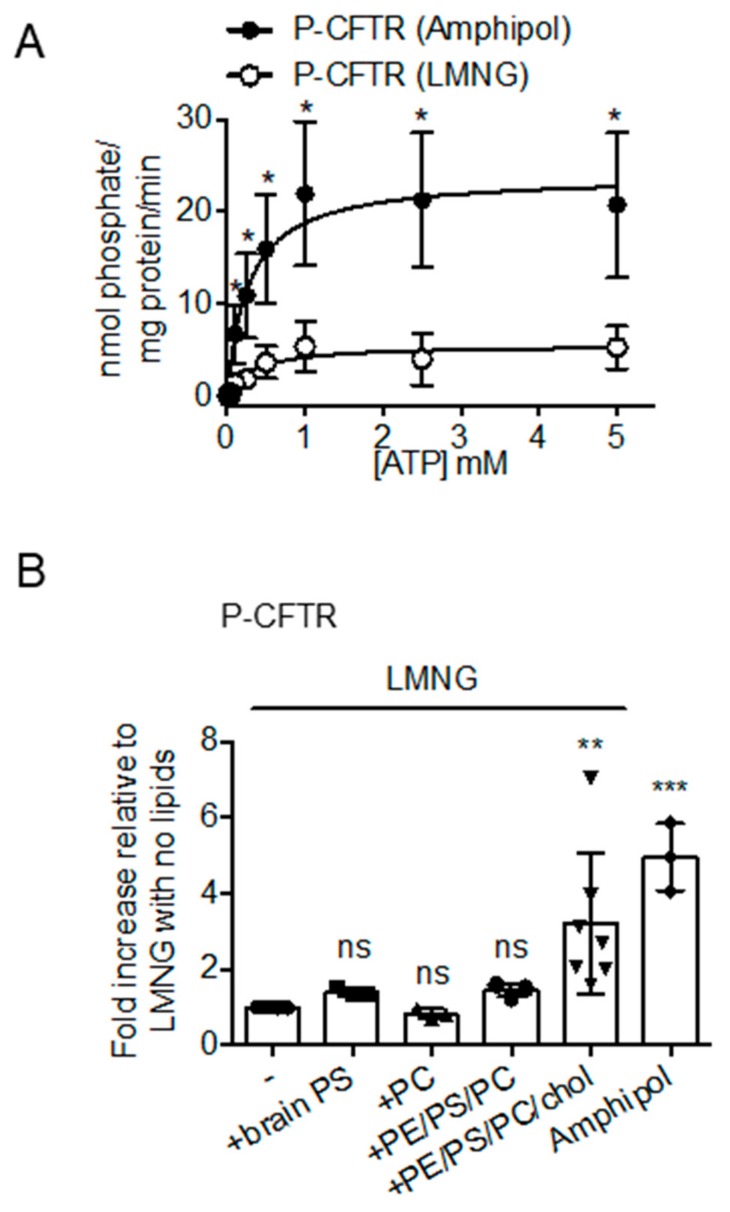
The specific ATPase activity of phosphorylated CFTR is higher in amphipol preparation than in detergent preparation and this difference is related to lipid associations of CFTR. (**A**) ATPase activity of P-CFTR in amphipol (closed circles) and in LMNG detergent (open circles) across ATP concentrations (mM) expressed as nmol phosphate/mg protein/min. The data is fitted to Michaelis-Menten curves and is presented as the mean ± SD (*n* = 3 biological replicates and *n* = 6 technical replicates). * *p* = 0.0026 at 0.1 mM ATP; * *p* = 0.0008 at 0.25 mM ATP; * *p* = 0.0006 at 0.5 mM ATP; * *p* = 0.0006 at 1.0 mM ATP; * *p* = 0.0003 at 2.5 mM ATP; * *p* = 0.0009 at 5.0 mM ATP; multiple t-tests. (**B**) The fold change of ATPase activity at 0.5 mM ATP relative to protein amounts of P-CFTR in LMNG detergent pre-treated with PC, brain PS or a mix of lipids PE/brain PS/egg PC/cholesterol (PE/PS/PC/chol) at 5:2:1:1 weight ratio for 1 h relative to P-CFTR in LMNG detergent. ATPase activity at 0.5 mM ATP of P-CFTR in amphipol was also compared as a reference. The data is presented as the mean ± SD (*n* = 4 biological replicates, *n* = 4 technical replicates for P-CFTR in LMNG and P-CFTR in LMNG pre-treated with PE/PS/PC/chol; *n* = 3 biological replicates, *n* = 3 technical replicates for P-CFTR in LMNG pre-treated with brain PS, PC and P-CFTR in amphipol). ns, not significant; ** *p* = 0.0012; **** *p* < 0.0001; One-way ANOVA with Dunnett’s multiple comparisons test comparing each condition to P-CFTR in LMNG detergent.

This study compared the specific ATPase activities of CFTR purified using amphipol or LMNG after PKA phosphorylation (P) to ensure that both preparations were maximally phosphorylated, a modification known to be important for CFTR function as an enzyme and a channel [4,5,6,38,39]. It was found that the K_m_ values of ATP were similar for the two samples: K_m_ of 0.27 ± 0.08 mM ATP for the purified P-CFTR in amphipol and K_m_ of 0.32 ± 0.14 mM ATP for the purified P-CFTR in LMNG detergent micelles (Figure 3A). Interestingly, the maximal ATPase activities, after normalization to protein amounts (Refer to CFTR quantification section in Materials and Methods for more details on the method), were significantly higher in the purified P-CFTR: amphipol complexes than in the purified P-CFTR: LMNG micelles (V_max_ of 23.90 ± 1.91 nmol phosphate/mg protein/min versus V_max_ of 5.54 ± 0.66 nmol phosphate/mg protein/min respectively, Figure 3A).

In order to determine the role of lipids in enhancing the ATPase activity of the amphipol preparation, this study tested the effect of re-introducing PC, PS, a mix of PE:PS:PC (5:2:1 ratio by weight), or a mix of PE:PS:PC:cholesterol (5:2:1:1 ratio by weight) in the LMNG preparation (Figure 3B). Interestingly, the cholesterol addition was required to induce a significant increase in ATPase activity of purified P-CFTR in LMNG detergent micelles (Figure 3B). These results suggest that the cholesterol interaction enhances catalytic activity of the CFTR molecules.

**Figure 4:**
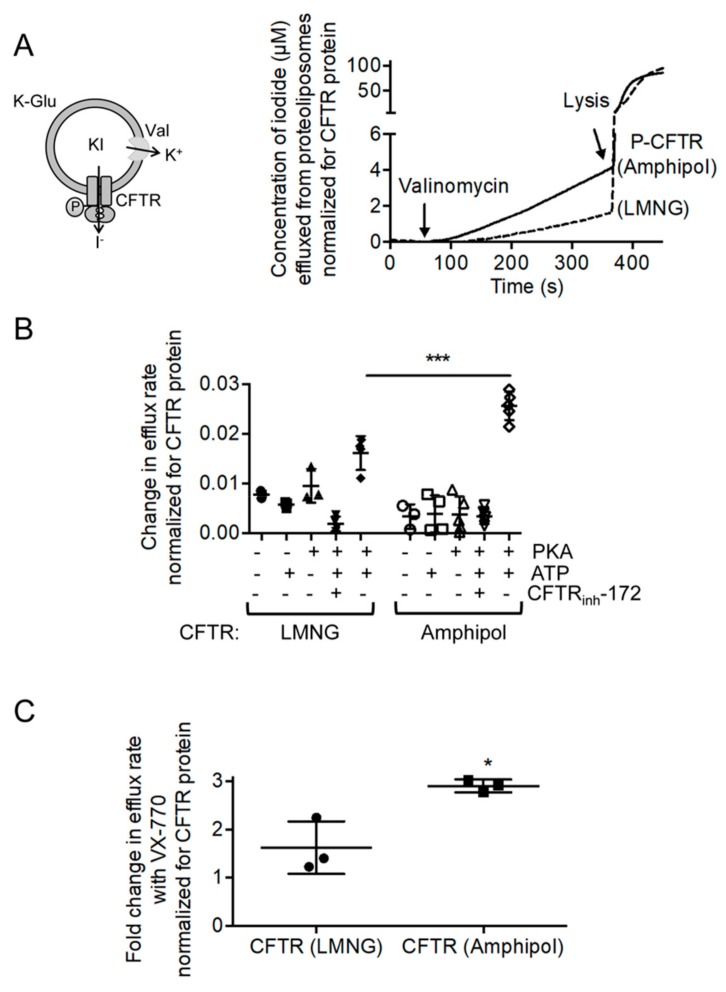
Amphipol protects channel-active conformation of CFTR. (**A**) A cartoon showing that proteoliposomes are loaded with potassium iodide (KI) on the inside with an equal concentration of potassium glutamate (K-Glu) on the outside. Valinomycin (Val), a potassium ionophore, provides a counterion pathway thereby facilitating iodide (I^−^) electrodiffusion via activated CFTR. The amount of I^−^ effluxed can be detected by an iodide-selective electrode. The representative iodide efflux traces showing concentrations of iodide (µM) effluxed from proteolipsomes normalized for CFTR protein (ng) reconstituted in liposomes. The iodide efflux traces of proteoliposomes reconstituted with P-CFTR pre-treated with Mg-ATP in amphipol and of proteoliposomes reconstituted with P-CFTR pre-treated with Mg-ATP in LMNG that were both treated with valinomycin and then lysed with Triton. (**B**) The change in iodide efflux rate normalized to CFTR amounts (ng) before and after valinomycin treatment of reconstituted P-CFTR in LMNG detergent and in amphipol with Mg-ATP along with negative controls: unphosphorylated CFTR with and without Mg-ATP, P-CFTR without Mg-ATP and P-CFTR with Mg-ATP treated with CFTR_inh_-172. The reconstituted P-CFTR in LMNG detergent and amphipol with Mg-ATP resulted in significant increases in the change of the slope of iodide efflux compared to negative controls. The reconstituted P-CFTR in amphipol with Mg-ATP resulted in a significantly higher change in the slope of iodide efflux compared to reconstituted P-CFTR in LMNG detergent with Mg-ATP. The data is presented as the mean ± SD (*n* = 3 biological replicates, *n* > 3 technical replicates). *** *p* < 0.0001; One-way ANOVA with Tukey’s multiple comparisons test. (**C**) The fold change in iodide efflux rate normalized for CFTR protein with 1 µM VX-770 compared to the vehicle shows that the potentiation effect of VX-770 was significantly higher in proteoliposomes reconstituted with P-CFTR in amphipol compared to proteoliposomes reconstituted with P-CFTR in LMNG detergent micelles. The data is presented as the mean ± SD (*n* = 3 biological replicates, *n* = 3 technical replicates). * *p* = 0.0355; paired t-test.

Next, this study determined whether the amphipol preparation enhanced the functional reconstitution of CFTR channel activity relative to the LMNG preparation. Each preparation was separately reconstituted into pre-formed 1,2-palmitoyl-oleoyl-sn-glycero-3-phosphocholine (POPC) liposomes. The anion electrodiffusion from liposomes normalized for the amount of CFTR inserted into liposomes were measured (Refer to CFTR quantification section in Materials and Methods for more details on the method) in order to determine the relative proportion of channel competent CFTR molecules from the two preparations [40,41]. Briefly, in this assay, CFTR protein in amphipols or LMNG micelles was reconstituted into POPC proteoliposomes with equal concentrations of potassium iodide inside and potassium glutamate outside of the liposomes to provide a driving force for iodide efflux and to maintain the osmolarity [40,41]. The addition of valinomycin, an ionophore that selectively facilitates potassium ion flux out of the proteoliposomes, alleviates the charge build up in the proteoliposomes and allows for iodide efflux through activated CFTR [40,41]. A schematic of this assay is shown in Figure 4A.

This study measured iodide efflux after valinomycin in addition to proteoliposomes containing either amphipol plus associated lipids or LMNG preparations of CFTR (Figure 4A). As expected for CFTR-mediated flux, the electrodiffusion of iodide was minimal for unphosphorylated CFTR or phosphorylated CFTR in the absence of Mg-ATP (Figure 4B) [40]. In addition, the CFTR inhibitor, CFTR_inh_-172, reduced the flux for the phosphorylated protein in the presence of Mg-ATP, pointing to the specificity of this function (Figure 4B) [40]. Together, the proteoliposomal flux assay reports on the phosphorylation- and ATP-dependent channel activity of CFTR. Importantly, a significantly higher rate of iodide efflux from proteoliposomes containing P-CFTR purified using amphipol relative to the proteoliposomes containing P-CFTR purified using LMNG (Figure 4B) was observed. Given that these proteoliposomal flux studies were controlled for the abundance of total CFTR reconstituted in proteoliposomes and the number of proteoliposomes (estimated by total trapped iodide, Supplementary Figure S1), these findings suggest that the lipids including cholesterol retained in the CFTR: amphipol complexes facilitate CFTR channel activation. However, the stoichiometry of cholesterol molecules that interact with CFTR following reconstitution into phospholipid liposomes remains unknown.

Ivacaftor (or VX-770) potentiates the regulated channel activity of Wt-CFTR and is approved as a therapeutic intervention for a number of disease-causing mutations [40,42,43,44]. We previously showed, using reconstituted CFTR purified using the detergent, *fos*-choline 14, that VX-770 directly modulates the channel opening of PKA phosphorylated CFTR [40]. Previous studies have suggested that the lipophilic compound, VX-770, interacts with the lipid bilayer and may bind at a CFTR: lipid interface [44,45,46]. In fact, the recent cryo-EM studies by Liu et al. modeled the binding site for VX-770 at a hinge region exposed to the detergent solvent [23]. Thus, it was reasoned that CFTR extracted with its associated lipids using amphipol would exhibit a different response to VX-770 than CFTR extracted with detergents. Now, this study shows that the fold increase in iodide electrodiffusion caused by VX-770 (1 μM) from proteoliposomes containing amphipol-extracted CFTR was approximately twice that measured for proteoliposomes containing LMNG-extracted protein (Figure 4C). Together with the ATPase activity assays, these findings suggest that the cholesterol extracted with CFTR by amphipols facilitates VX-770 binding and/or the conformational changes required for its potentiation.

## 3. Discussion

A novel, detergent-free method for CFTR purification that retains associated lipids revealed an important role for cholesterol in modifying CFTR activity. CFTR was co-purified with phospholipids and cholesterol using amphipol A8-35 and this complex exhibited higher rates of specific ATPase activity and regulated anion channel activity than CFTR in detergent micelles. This study showed that addition of cholesterol, together with phospholipids, to detergent-purified CFTR enhanced its ATPase function. Furthermore, the anion channel activity of amphipol-purified CFTR, containing cholesterol plus phospholipids exhibited a better response to the drug, ivacaftor (VX-770), relative to that measured for detergent purified CFTR after reconstitution, suggesting that the efficacy of VX-770 in potentiating CFTR is also modulated by lipids [45,46].

Although CFTR purified in detergents can be functionally reconstituted as a phosphorylated and ATP-regulated anion channel in phospholipid liposomes [8,13,38,47,48], it was shown that its reconstitution following amphipol purification confers superior activity. The proportion of purified CFTR that can be functionally reconstituted as an anion channel was not routinely defined in other published purification protocols, including those used to generate the six cryo-EM structures [19,20,21,22,23]. Hence, a systematic comparison of the fidelity of functional reconstitution of CFTR, purified using different detergents is not possible. However, the current work directly compared detergent (LMNG)-purified CFTR versus amphipol-purified CFTR and this study showed that the magnitude of CFTR mediated anion channel activation, normalized for CFTR protein mass, was approximately two-fold greater for the amphipol preparation. This difference may reflect either an increase in the number of functionally reconstituted CFTR molecules, or an acute increase in the intrinsic activity of each CFTR molecule if its annulus of associated lipids is preserved. The former option is plausible, as the percent of total CFTR protein that is catalytically active after purification using LMNG is expected to be low based on the studies shown in Figure 1 where there was a displacement of the peak ATPase versus CFTR protein abundance, even for the amphipol-purified protein. Given the acute effect of cholesterol addition to the ATPase activity of CFTR after purification in detergent micelles, it was suggested that cholesterol and phospholipids that are co-purified with CFTR by amphipols can directly modulate the intrinsic channel activity of CFTR molecules in each CFTR: amphipol: cholesterol-containing proteoliposome.

Comprehensive reviews describing putative mechanisms underlying ATP dependent gating of CFTR have been published recently [10,11,49,50]. In brief, the form of the CFTR protein that is phosphorylated at key PKA consensus sites and has ATP bound at both ATP binding sites exhibits the highest open probability. This open state is favored by ATP binding and disruption of ATP turnover by mutations of the dominant catalytic base, E1371 [8,10,11,51]. Therefore, our observation that lipids, including cholesterol, increase both ATPase activity and channel opening, may be explained by an increase in ATP binding to the catalytic site in CFTR. In our opinion, the development of single molecule ATP binding and ATPase activity assays are required to complement single channel electrophysiological studies in order to add clarity to our understanding of the role of specific lipids in ATPase and channel gating activities of CFTR.

This study showed previously that the channel activity of detergent-purified CFTR was potentiated by ivacaftor (VX-770) following its reconstitution into liposomes, supporting the claim that VX-770 acts via direct binding to CFTR [40]. Another study recently reported the interaction of VX-770 with detergent-solubilized CFTR using hydrogen/deuterium exchange [52]. This method showed that there were multiple regions in CFTR that underwent changes in conformation after VX-770 interaction.

Hwang and colleagues used drug-free, cryo-EM structures to identify putative VX-770 binding sites using in silico docking methods [53]. Based on this data, it was postulated that there are two putative binding sites for VX-770 lying at the interface of the two membrane spanning domains of CFTR. The location of these sites in the membrane domains are consistent with the hydrophobic nature of VX-770 (log*P* score of 5.76) [45,54] and the observation that VX-770 has been found to insert into the lipid bilayer [45].

The ivacaftor binding site in CFTR was recently modeled in the cryo-EM structure of the CFTR: drug complex [23]. In that study, ivacaftor was modeled at the discontinuity in transmembrane spanning helix 8 (tm8), a region that the authors previously showed to be important for channel gating [20,22] and corresponded to Site 2 for ivacaftor binding that was identified by the Hwang group [53]. Interestingly, in this cryo-EM model, 40% of the ivacaftor molecule interacts with the CFTR protein at the elbow-like joint via hydrogen bond interaction with residue S308 and interaction with aromatic residues: F312 and F931. On the other hand, 60% of the ivacaftor molecule projected into the detergent and the authors speculated that more than half of this drug interacted with lipids. This proposal is consistent with our findings that the lipid environment modulates the activity of ivacaftor as a CFTR potentiator.

Cholesterol is present at the plasma membrane and interacts with a population of CFTR in microdomains known as lipid rafts [55,56]. The interaction of CFTR and cholesterol regulates the confinement and dynamics of CFTR at the cell surface [55,56]. Prior to this study, cholesterol was only thought to act indirectly rather than directly to regulate the function of CFTR. According to a recent study by Hanrahan and colleagues, secretagogues, like vasoactive intestinal peptide acted to promote association of CFTR with cholesterol-containing clusters on the apical surface of respiratory epithelial cells [57]. This clustering restricts the lateral mobility of CFTR, thereby enabling its efficient incorporation into macromolecular complexes important for phosphorylation-dependent regulation and integration with larger, ceramide rich platforms [57].

The current study is the first to show a direct role for cholesterol in augmenting the ATPase activity of CFTR. Hence, this study proposes that CFTR localization to lipid rafts may modify CFTR activity, both by regulating its proximity to macromolecular signaling complexes and also by enhancing its intrinsic function.

Interestingly, the addition of phospholipids (PE, PC and PS) back to the delipidated CFTR was ineffective in restoring ATPase activity in contrast to the previously reported stimulatory effect of PS [33]. The positive effect of PS was previously measured in CFTR solubilized using decyl maltose neopentyl glycol (MNG10) [33,58] rather than LMNG and this may account for the difference in our results.

Our studies support a growing literature showing that lipid binding can specifically enhance channel activity. This has been reported for voltage-gated potassium and sodium channels [59,60,61]. The evidence supporting a role for lipids in modulating drug binding and efficacy is also mounting in structural studies of membrane proteins including the voltage-gated calcium (Ca_V_Ab) channel [62]. The new methods for purifying CFTR together with its interacting lipids may facilitate a deeper understanding of the regulation of its gate and potentiation by drugs in the clinic.

## 4. Materials and Methods

### 4.1. Generation of FLAG-CFTR-His Construct

The N-terminal DYKDDDDK (FLAG) tag was introduced on the CFTR-His construct (with a C-terminal 10× His-tag) on the plasmid DNA containing WT-CFTR cDNA (in pcDNA3.1) as the template using the KAPA HiFi HotStart Ready Mix (KAPA Biosystems, Wilmington, MA, USA), primers (Forward: 5′- GAG ATG GAT TAT AAA GAT GAT GAT G -3′, Reverse: 5′- CAT CAT CAT CTT TAT AAT CCA TCT C -3′) and polymerase chain reaction. The construct was validated by DNA sequencing (TCAG, Toronto, Ontario, Canada) and amplified by transformation and plasmid maxi-prep (Qiagen, Hilden, Germany). The high-quality (260/280 nm ratio of 1.8 and higher) plasmid DNA was concentrated to a final concentration of 1 μg/μL.

### 4.2. Expression of FLAG-CFTR-His Construct in HEK293F Cells and Generation of Crude Membranes

The FLAG-CFTR-His construct was transiently transfected in HEK293F (Thermo Fisher Scientific) suspension cell line capable of complex N-glycosylation. The DNA at 50 μg was mixed in a 1:1 ratio with transfection reagent FectoPRO^®^ (Polyplus, Berkeley, CA, USA) and added to each 200 mL suspension culture at 0.8 × 10^6^ cells per mL, as previously described [63,64]. The cells were grown at 37 °C shaking at 180 rpm with 8% CO_2_ for 24 h. The next day, the cells were treated with 10 mM sodium butyrate (Sigma-Aldrich, St. Louis, MO, USA) at 37 °C shaking for 24 h. The HEK293F cells transfected with FLAG-CFTR-His were collected and spun down at 6000 rpm at 4 °C for 30 min. The fresh or frozen cell pellet generated from a 600 mL cell suspension was re-suspended in 40 mL of phosphate-buffered saline (PBS, Wisent Inc., Saint-Jean Baptiste, Quebec, Canada) containing one tablet of cOmplete, EDTA-free protease inhibitor (Roche, Mannheim, Germany). The cells were lysed using a Emulsiflex C3 high pressure homogenizer (Avestin, Ottawa, Ontario, Canada) with at least 5 passages during the lysing process. The cell debris was spun down at 1000× *g* at 4 °C for 15 min. The supernatant from this spin was further spun down at 100,000× *g* at 4 °C for 2 h. The pelleted crude membrane was re-suspended in 20 mL of PBS containing 0.1 mM DDM (BioShop, Burlington, ON, Canada) and protease inhibitor (Roche). The syringes with 21G and 27G needles (BD, Franklin Lakes, NJ, USA) were used to disaggregate the pellet. The DDM-treated membranes were incubated on ice for 10 min. Any heavy particulate matter that came down was discarded before spinning the membrane suspension at 100,000× *g* at 4 °C for 2 h. The extracted crude membrane pellet was again re-suspended with the aid of the syringe and previously described needles in 20 mL of 25 mM HEPES, 100 mM NaCl buffer with protease inhibitor (Roche) and then aliquoted. The total protein concentration of the crude membranes was determined by Bradford assay [65] and the presence of CFTR was confirmed by immunoblotting.

### 4.3. Purification of FLAG-CFTR-His in Amphipol or Detergent

The crude membranes were solubilized to a final concentration of 2 mg/mL with 1% LMNG (Anatrace, Maumee, OH, USA) and 0.2% cholesterol hemisuccinate (Anatrace) in buffer A (20 mM Tris-HCl pH 7.5, 2 mM MgCl_2_, 200 mM NaCl, 20% glycerol, and 2 mM DTT, with protease inhibitor [Roche]) as previously described [19,20,21,22] or with 3% (*w/v*) amphipol A8-35 (Anatrace) in buffer B (25 mM HEPES pH 8, 100 mM NaCl with protease inhibitor [Roche]) at 4 °C for 2 h with shaking. The insoluble fractions were removed by ultracentrifugation at 45,000 rpm at 4 °C for 1 h. Anti-FLAG^®^ M2 magnetic beads (Sigma-Aldrich) were washed with corresponding buffers (buffer A with 0.025% LMNG or buffer B with 0.025% amphipol) using a magnetic rack (New England Biolabs, Ipswich, MA, USA) for a total of 3 times. The soluble fractions were incubated with anti-FLAG^®^ M2 magnetic beads at 4 °C overnight with shaking. The unbound proteins were removed the next day with a magnetic rack (New England Biolabs) and anti-FLAG^®^ M2 magnetic beads (Sigma-Aldrich) were washed 5 times with corresponding buffers. If phosphorylation was required, the anti-FLAG^®^ M2 magnetic beads (Sigma-Aldrich) were treated with a phosphorylation cocktail (10,000 U of PKA [New England Biolabs] and 5 mM Mg-ATP [Sigma-Aldrich] in buffer A with 0.025% LMNG or buffer B with 0.025% amphipol) at room temperature for 1 h with shaking. The phosphorylation cocktail was removed by a magnetic rack and the anti-FLAG^®^ M2 magnetic beads were washed again for 5 times with the corresponding buffers. The FLAG-CFTR-His protein was eluted with 200 μg/mL FLAG^®^ peptide (DYKDDDDK, Sigma-Aldrich) in buffer A with 0.025% LMNG or buffer B with 0.025% amphipol using the magnetic rack after 45 min incubation with shaking at 4 °C. The purified protein at 500 μL was loaded on a Superose 6 Increase 10/300 column (GE Healthcare) to separate the protein based on its size. The purity of purified CFTR was determined by densitometry of the CFTR bands compared to other bands present on the 4–12% silver stained gels (SDS-PAGE) using the Image Studio^TM^ Lite software.

### 4.4. CFTR Quantification

The crude membranes at 2.6 mg were solubilized in amphipol and CFTR was purified in amphipol as described above to generate 400 μL of eluted standard in the presence of FLAG^®^ peptide. An aliquot of standard (100 μL) was subjected to amino acid analysis with the Waters Pico-Tag System (Waters ACQUITY UPLC, Milford, MA, USA). (Refer to Supplementary Figure S2 and Supplementary Table S1 for complete results of amino acid analysis of the purified CFTR standard in amphipol.) Alanine (1015.112 pmoles were present in the 100 μL standard) was chosen to determine the concentration of CFTR standard as amino acid hydrolysis with various solvents resulted in 100% recovery of alanine [66] and there were no alanine residues in the FLAG^®^ peptide that was also present in the standard. The calculation of the concentration of the CFTR standard is as follows:

(1) $$\frac{weight\;Ala}{weight\;CFTR}=\frac{number\;of\;Ala*\left(MW\;Ala\right)}{MW\;CFTR}$$

(2) $$\frac{72065\;pg}{weight\;CFTR}=\frac{83*71\;pg/pmol}{170507.57\;pg/pmol}$$

The weight of CFTR = 208,512,269 pg or 2085.12269 ng; the volume used for amino acid analysis = 100 µL; the concentration of CFTR standard = 2085.12269 ng / 100 µL = 20.85 ng/µL.

The remaining standard was aliquoted and stored at −80 °C. When required, various nanogram amounts of the standard were run with purified protein samples of unknown amounts on SDS-PAGE and subjected to immunoblotting. The immunoblot bands were analyzed with Image Studio^TM^ Lite software and densitometry of unknowns were interpolated on the standard curve in GraphPad Prism. (Refer to Supplementary Figure S3 for example of standard curve for concentration determination.)

### 4.5. Phospholipid Assay

The purified CFTR in amphipol, purified CFTR in LMNG and the appropriate controls were subjected to a 96-well phospholipid assay kit according to the manufacturer’s protocol (Cat. no. MAK122, Sigma-Aldrich). Fluorescence was read at an excitation of 530 nm and the emission of 585 nm on the SpectraMax i3X plate reader (Molecular Devices).

### 4.6. Lipid TLC

Lipid-containing samples were extracted as previously described [67]. The lipid-containing organic phase was first dried with sodium sulfate for 1 h, filtered and concentrated under a stream of argon to a volume of 50 μL. The silica gel plates (Analtech Inc.) were first activated at 160 °C for 1 h before the sample application. The samples (10 μL) and polar lipid standard (2 μL, Matreya LLC) were spotted and developed using chloroform: methanol: water (65:25:4) as the solvent system. The visualization of the lipids was conducted as previously described [67].

### 4.7. ATPase Assay–ATP Dose Response

The purified protein (approximately 50–200 ng in 25 μL) was incubated with 50 μL of various concentrations of Na-ATP (Sigma-Aldrich) solubilized in 10 mM HEPES, 100 mM NaCl, 5 mM MgCl_2_, 1 mM DDM, pH 7.4 buffer at 37 °C for 2 h. A colorimetric assay was applied as previously described [68] to detect phosphate at absorbance 800 nm on the SpectraMax i3X plate reader (Molecular Devices). The values were subtracted from the controls (amphipol or LMNG buffer with various concentrations of ATP), converted to nmol phosphate by interpolation of the phosphate standard curve on GraphPad Prism and then to nmol phosphate/mg protein/min after CFTR quantification as previously described. The values were fit on Michaelis-Menten curves with GraphPad Prism to determine the K_m_ and V_max_ values of ATPase activity.

### 4.8. ATPase Assay–Re-Introducing Lipids to Purified CFTR in LMNG Detergent Micelles

The egg PC, porcine brain PS, a mix of PE/brain PS/egg PC in a 5:2:1 (*w/v*) ratio, or a mix of PE/brain PS/egg PC/cholesterol in a 5:2:1:1 (*w/v*) ratio (all lipids were in chloroform from Avanti^®^ Polar Lipids, Inc.) were dried in a glass tube under argon gas. The dried lipids were re-suspended in 25 mM HEPES, 100 mM NaCl, pH 8.0 buffer with sonication to a stock concentration of 10 mg/mL. The re-suspended lipid (100 μg in 10 μL) was added to the purified protein (approximately 50–200 ng in 25 μL) in a 96-well plate along with controls: (1) 10 μL of 25 mM HEPES, 100 mM NaCl, pH 8.0 buffer was added to 25 μL of purified protein; (2) 10 μL of re-suspended lipid was added to 25 μL of buffer A with 0.025% LMNG; (3) 10 μL of 25 mM HEPES, 100 mM NaCl, pH 8.0 buffer was added to 25 μL buffer A with 0.025% LMNG. The protein with lipids and controls were incubated at 1 h at room temperature. The Na-ATP (40 μL of 0.9375 mM stock concentration to a final concentration of 0.5 mM Na-ATP) solubilized in 10 mM HEPES, 100 mM NaCl, 5 mM MgCl_2_, 1 mM DDM, pH 7.4 buffer was added to the mix and the reaction was incubated at 37 °C for 2 h. The colorimetric assay was applied as described above.

### 4.9. Iodide Efflux

The CFTR protein in amphipol was incubated with 5 mM DDM during the elution step with a FLAG^®^ peptide prior to reconstitution, whereas the protein in LMNG remained as is. The purified protein (approximately 0.4 μg) was reconstituted in 5 mg of POPC (Avanti^®^ Polar Lipids, Inc.) and subjected to iodide efflux as previously described [40,41].

## Supplementary Materials

The following are available online at https://www.mdpi.com/2073-4409/8/8/804/s1, **Figure S1**: Trapped iodide is similar for the two reconstitutions, reconstitution of LMNG purified CFTR and amphipol purified CFTR. Bars correspond to iodide measurements before valinomycin addition and after detergent lysis normalized to CFTR amounts. The data is presented as mean ± SD (*n* > 3 biological replicates and *n* > 3 technical replicates). *p* = 0.9974; paired t-test. **Figure S2**: Results of amino acid analysis of CFTR standard. (**a**) Chromatogram of all the amino acids present in CFTR standard. (**b**) Scaled chromatogram to magnify the smaller peaks. **Figure S3**: Representative immunoblot and standard curve applied for CFTR quantification. (**a**) Immunoblot of CFTR standard at increasing amounts (nanograms). (**b**) Standard curve of (**a**) that is used for interpolation of unknown purified protein. **Table S1**: Summary table of amino acid analysis results of CFTR standard. Table shows the amino acid (3-letter code), retention time (RT) in minutes of when the amino acid shows up, the area under the peak and the corresponding amount of the amino acid in picomoles (pmoles) in a 100 µL purified CFTR standard. **Table S2**: Variables for determining concentration of CFTR standard.

## Abbreviations

CFTR: cystic fibrosis transmembrane conductance regulator; ABC, ATP-binding cassette; PKA, protein kinase A, R, regulatory; NBD, nucleotide binding domain; EM, electron microscopy; TMD, transmembrane domain; TM, transmembrane segments; SEC, size exclusion chromatography; LMNG, lauryl maltose neopentyl glycol; PC, phosphatidylcholine; PE, phosphatidylethanolamine; TLC, lipid thin-layer chromatography; PS, phosphatidylserine; POPC, 1,2-palmitoyl-oleoyl-sn-glycero-3-phosphocholine
