# Supplementary material for: Cholesterol Interaction Directly Enhances Intrinsic Activity of the Cystic Fibrosis Transmembrane Conductance Regulator (CFTR)
Source: Cells. 2019 Jul 31;8(8):804. doi: 10.3390/cells8080804 (PMC6721619; doi:10.3390/cells8080804)
Supplement: Supplementary file 1 [file cells-08-00804-s001.pdf]

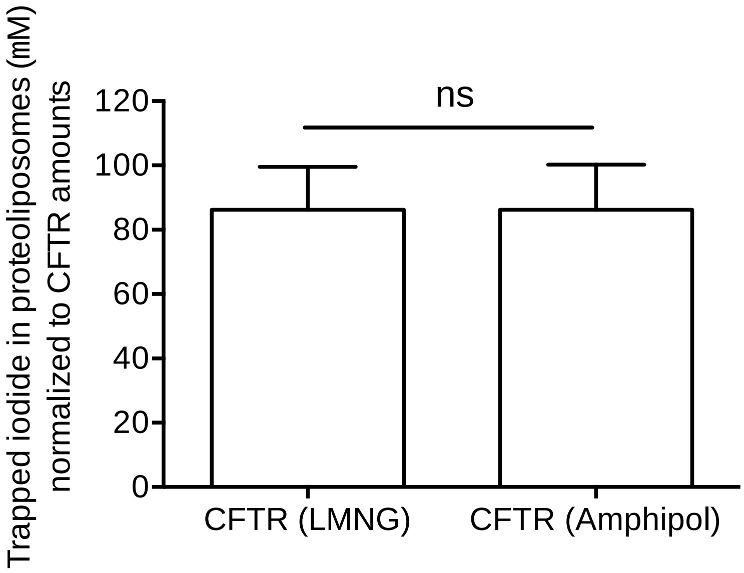

Supplementary Figure 1: Trapped iodide is similar for the two reconstitutions, reconstitution of LMNG purified CFTR and amphipol purified CFTR. Bars correspond to iodide measurements before valinomycin addition and after detergent lysis normalized to CFTR amounts. The data is presented as mean ± SD (n > 3 biological replicates and n > 3 technical replicates). $P = 0.9974$; paired t-test.

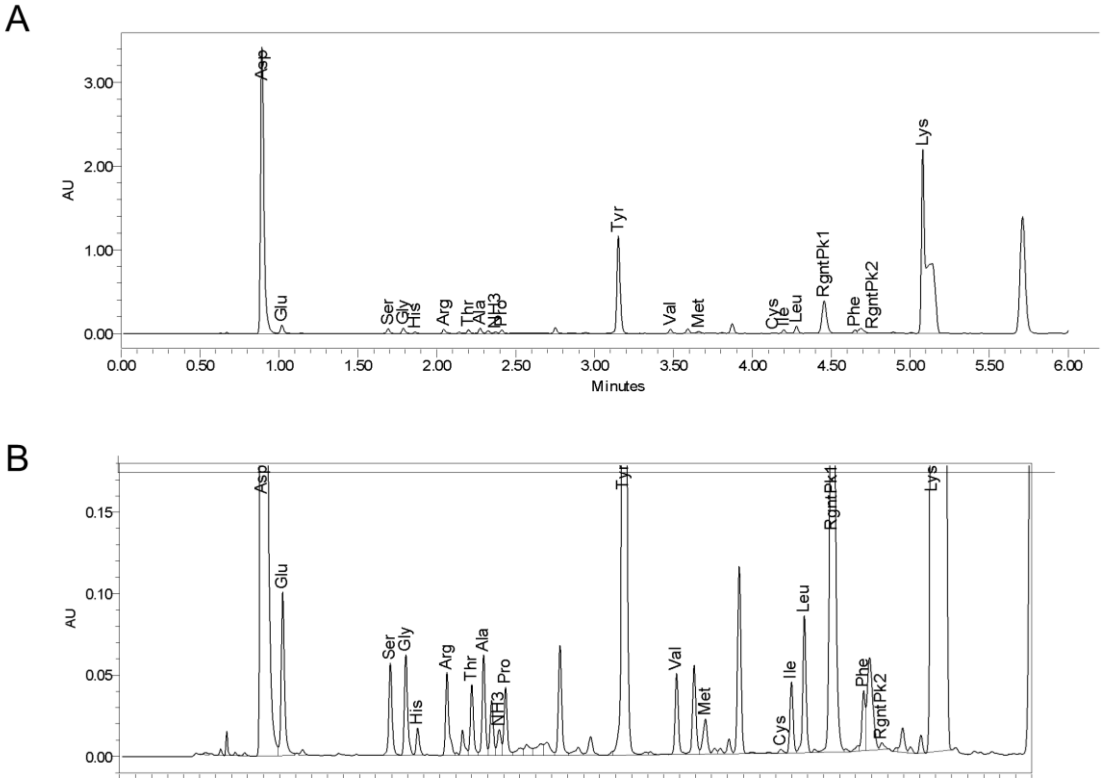

Supplementary Figure 2: Results of amino acid analysis of CFTR standard. (a) Chromatogram of all the amino acids present in CFTR standard. (b) Scaled chromatogram to magnify the smaller peaks.

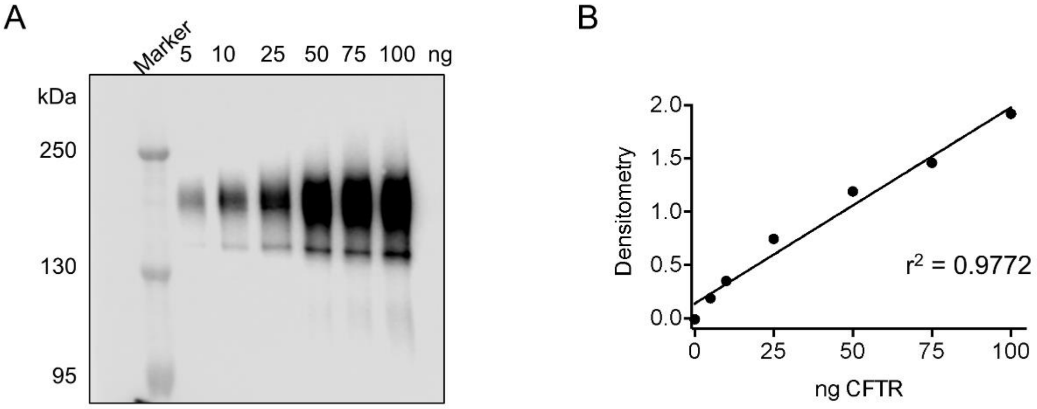

Supplementary Figure 3: Representative immunoblot and standard curve applied for CFTR quantification. (a) Immunoblot of CFTR standard at increasing amounts (nanograms). (b) Standard curve of (a) that is used for interpolation of unknown purified protein.

Supplementary Table 1: Summary table of amino acid analysis results of CFTR standard. Table shows the amino acid (3-letter code), retention time (RT) in minutes of when the amino acid shows up, the area under the peak and the corresponding amount of the amino acid in picomoles (pmoles) in a 100 µl purified CFTR standard.

**Amino acid**	**RT**	**Area**	**Amount (pmoles)**
Asp	0.892	5099166	66398.567
Glu	1.019	147780	2021.338
Ser	1.692	79642	1035.873
Gly	1.788	86381	1139.691
His	1.862	22241	289.816
Arg	2.045	74065	947.141
Thr	2.201	59033	747.984
Ala	2.275	83745	1015.112
Pro	2.412	59190	713.162
Tyr	3.150	1680122	19755.248
Val	3.480	70278	882.894
Met	3.661	41282	496.935
Cys	4.132	4485	37.648
Ile	4.198	60431	686.995
Leu	4.279	124398	1497.008
Phe	4.650	53427	618.165
Lys	5.079	5901164	38071.770

Supplementary Table 2: Variables for determining concentration of CFTR standard.

**Variable**	**Value**
MW Ala	89.0935 – 18 (for peptide bond)<br>= 71 g/mol or pg/pmole
weight Ala	1015.12 pmoles (from chromatogram)<br>1015.12 pmoles x 71.0935 pg/pmole<br>= 72065 pg
number of Ala in CFTR	83
